# Identification of microbial diversity in buried ivory soil at the Sanxingdui site in Guanghan City, China, using high-throughput sequencing

**DOI:** 10.3389/fmicb.2024.1384650

**Published:** 2024-05-30

**Authors:** Siyu Sun, Zhe Xu, Mengjia Ren, Sifan Li, Zhenbin Xie, Yanbing Luo, Yongqiang Tian

**Affiliations:** ^1^College of Biomass Science and Engineering, Sichuan University, Chengdu, China; ^2^School of History and Culture, National Center for Experimental Archaeology Education, Sichuan University, Chengdu, China; ^3^Sichuan Provincial Cultural Relics and Archaeology Research, Chengdu, China

**Keywords:** Sanxingdui site, ivory decomposition, high-throughput sequencing, 16S rRNA, ITS, community

## Abstract

**Introduction:**

The Sanxingdui Site in Guanghan City, Sichuan Province, China, is one of the precious heritage sites of the ancient Chinese civilization. Archaeological work at Sanxingdui is of great significance in clarifying the origins and main contents of the ancient Shu culture and the Yangtze River civilization. Since the 1920s, archaeologists have conducted extensive excavations and research at the site, with particular attention given to the large number of ivory artifacts unearthed. However, the buried ivory is influenced by soil pH, temperature, humidity, and other physical and chemical factors, along with the potential impact of microbial activities that may lead to the corrosion and decomposition of ivory. By understanding the types and activities of microorganisms, appropriate measures can be taken to protect and preserve cultural relics.

**Methods:**

Multi-point sampling of soil samples around the ivory of the three sacrificial pits at the Sanxingdui site was carried out, and strict aseptic operation was carried out during the sampling process. Subsequently, the microbial community structure and diversity in the buried ivory soil of Sanxingdui site were identified and analyzed by Illumina high-throughput sequencing technology.

**Results:**

16S rRNA and internal transcribed spacer sequence analysis revealed significant differences in the soil microbial community structure among different sacrificial pits. The dominant bacterial phyla were the Proteobacteria, GAL15, Actinobacteriota, Bacteroidota, and Methylomirabilota. The dominant fungal phyla were Ascomycota, Mortierellomhcota, and Basidiomycota. Most dominant bacterial and fungal communities play an indispensable role in the ivory corrosion mechanism, promoting the decay and decomposition process through various means such as decomposing organic matter and producing acidic substances.

**Discussion:**

It is particularly important to take a series of measures to control microbial activity to effectively protect ivory. Our preliminary study of the mechanism of action of microorganisms on ivory in a buried environment provides a scientific basis to prevent and protect against microbial degradation in ancient ivory unearthed in Sanxingdui. Following the research results, suitable antibacterial agents tailored to the preservation environment and microbial characteristics of ancient ivory can be prepared. Ensure that the selected antibacterial agents meet safety and effectiveness requirements to maximize protection against microbial degradation of ancient ivory.

## 1 Introduction

Microbial damage to cultural relics is an important issue of concern in the field of archaeological heritage conservation. Microbial activity-induced degradation in archaeological artifacts not only damages the physical structure and chemical composition of the artifacts themselves but may also affect their preservation status, aesthetic value, and significance to historical culture. Microbial infestation is widespread across various types of cultural relics, posing formidable challenges to relic conservation efforts. For instance, in ancient books and paper artifacts, microbes can form mold spots, leading to paper deterioration and damage (Sterflinger and Pinzari, 2012). On paintings, they may cause pigment fading, surface contamination, or the formation of spots (Ciferri, 1999). Ceramic artifacts are susceptible to microbial surface growth, resulting in the formation of mucous layers or cracks (Zhao et al., 2023). Furthermore, relics in soil sites are influenced by soil microbes, which may cause issues such as acidification and decay (Yang et al., 2020). However, due to the significantly smaller quantity of excavated ivory compared to other types of artifacts, research on the microbial impact on ivory artifacts is relatively limited.

Ivory is a unique hard animal tissue that is widely used in sculpture and art production owing to its hard texture and good luster (Conard, 2003). These works are masterpieces of craftsmanship and vivid manifestations of ancient cultural and social values, reflecting the prosperity and evolution of ancient cultures. In some cultures, ivory is regarded as a luxury, and people with ivory products are considered to have noble social status (Blier, 1993). Consequently, ivory products convey social class and identity along with representing symbols of material civilization. In recent years, advances in technology have gradually diversified research methods for ancient ivory to include microstructure analysis (Yamaguchi et al., 2001), isotope analysis (Britton et al., 2015), carbon-14 dating analysis (Alkass et al., 2011), DNA analysis (Orlando et al., 2021), morphological and anatomical research (Hart et al., 2020), and microbiological research (Dupont et al., 2007). Microstructural analysis revealed the ivory growth process, and isotope analysis revealed the climate and vegetation characteristics of the ancient ecological environment. Through the carbon-14 dating method, the age of ancient ivory can be accurately delineated, providing key information on the historical background of cultural relics. DNA analysis enables the identification of the species of ivory and investigation of the genetic relationship and genetic variation among individual ivory. Morphological and anatomical studies have further complemented our understanding of the whole picture of ivory. Microbiological studies have revealed the existence and influence of microorganisms during the burial of ancient ivory, providing key information for the protection and restoration of cultural relics. Integrating these multilevel research methods facilitates tracing of the natural history and cultural significance of ancient ivory, providing profound insights into anthropology, archaeology, ecology, and other fields.

As is well known, ivory is mainly composed of 70% inorganic components (primarily hydroxyapatite) and 30% organic components (primarily proteins) (Locke, 2008; Virág, 2012; Gong et al., 2019). However, long-term underground burial has deteriorated its appearance and internal structure. Notably, the composition of ivory, particularly its organic components, provides an ideal growth environment for microorganisms. The acidic substances and enzymes produced by microbial metabolism in the buried environment have a corrosive effect on ivory, resulting in its dissolution and the release of minerals, which has a non-negligible effect on the long-term stable preservation of ivory (Yu et al., 2023; Zhang et al., 2023, 2024). This corrosion process may accelerate ivory deterioration and destroy its original firmness and structural integrity. Therefore, the potential negative effects of microbial activity in a buried environment should be fully considered when studying the protection and preservation of ivory.

The Sanxingdui site is located in Sanxing Village, Sanxingdui Town, Guanghan City, Sichuan Province, China. It is one of the greatest archaeological discoveries of mankind in the 20th century, dating back 5,000–3,000 years. This site provides important archaeological materials for the in-depth study of ancient Shu culture and key information to understand the development process of Chinese civilization in Southwest China (Hua, 2013). In archaeological excavations, many cultural relics, such as stones, pottery, jade, and bronze wares were unearthed, besides many ivory wares (Li et al., 2023b). More than 80 ivories were unearthed from sacrificial pits no. 1 and no. 2, which were excavated in 1986. Additionally, new sacrificial pits were recently discovered. Among them, many relics were unearthed from pit no. 3 (K3), which is in the middle of all the sacrificial pit areas. The ivory in pit no. 4 (K4) was buried in the whole root, and the preservations condition were poor. Furthermore, nearly all the ivory underwent exposure to fire, and the upper layer of the ivory was filled with extensive black ashes, with bamboo as the main component. The eighth pit (K8) contained the largest number of ivories, and a layer of bronze artifacts was buried at the bottom of the ivory layer. As of September 2022, approximately 200, 104, and 47 complete ivories were unearthed in K8, K3, and K4, respectively. This series of discoveries provide valuable information for comprehensive analysis of the cultural heritage, history, and social structure of the Sanxingdui Site.

The ivories unearthed from the Sanxingdui Site have different degrees of decay, saturation, incompleteness, discoloration, surface pulverization, and mildew. The diversity of microorganisms surrounding the ivory has not been comprehensively studied using modern genomic methods. It is often difficult to analyze all possible microorganisms using traditional microbial research methods owing to the limitations of culture conditions. In contrast, high-throughput sequencing can capture microbial information on the surface or inside cultural relics with high sensitivity and comprehensiveness without relying on specific culture conditions (Pallen et al., 2010; Reuter et al., 2015; Ding et al., 2020). This advantage allows us to better understand the role of microorganisms in the corrosion of cultural relics, particularly in the discovery of microorganisms that are difficult to cultivate in the laboratory.

Therefore, in order to explore the effects of microorganisms on ivory in the burial environment, this study used high-throughput sequencing technology to analyze the diversity and composition of bacterial and fungal communities in 30 soil samples collected from the sacrificial pits at Sanxingdui Site. Through in-depth understanding of the potential hazards of microorganisms on ivory, we not only provide a reference for the scientific and effective protection of ancient ivory unearthed from Sanxingdui and the formulation of related protection programs, but also provide guidance for the protection of other cultural relics in similar environments. This contributes to the safeguarding of cultural heritage worldwide and promotes the inheritance and development of human civilization.

## 2 Materials and methods

### 2.1 Sample collection

**Figure 1:**
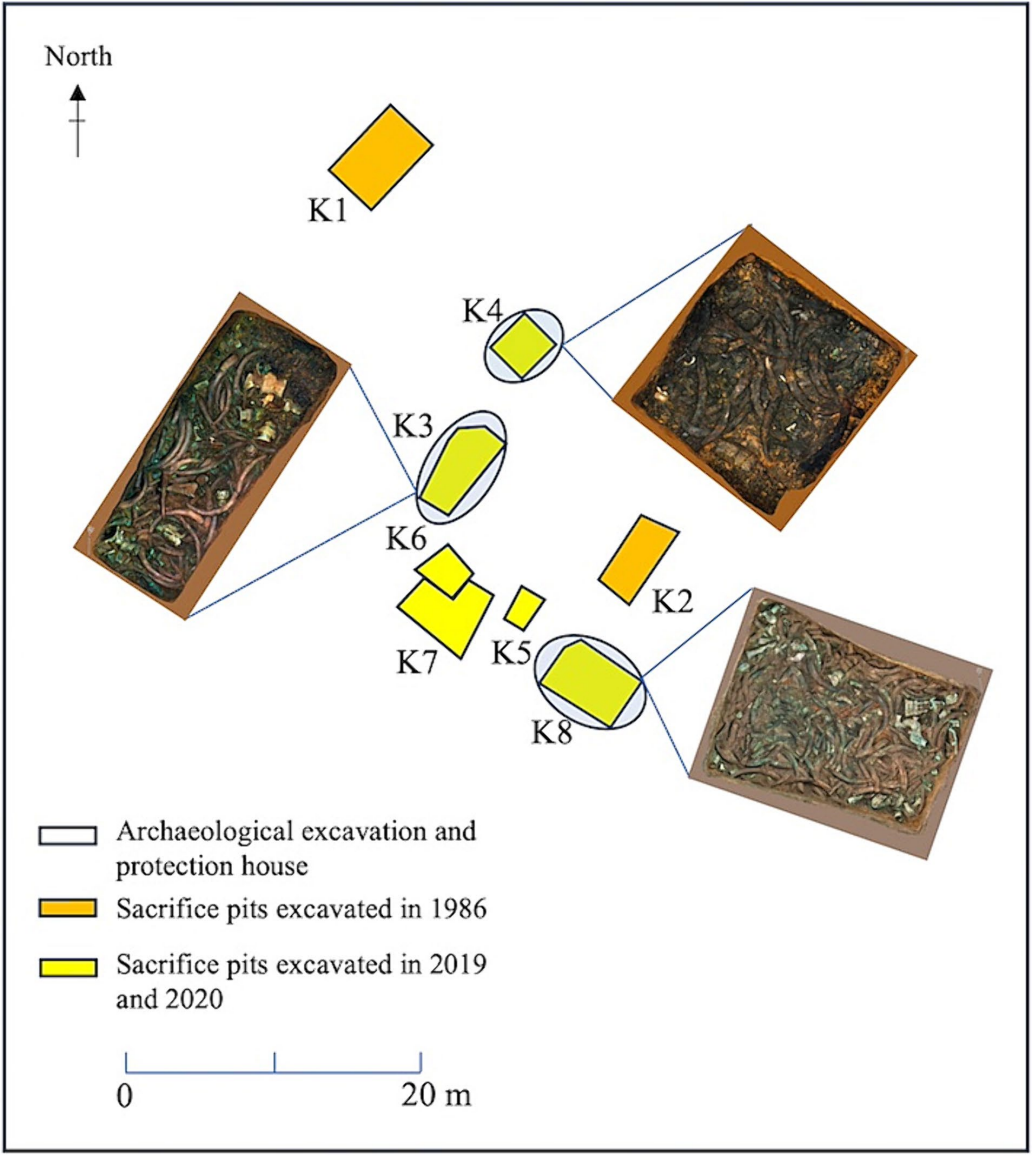
Distribution of sacrificial pits and soil sample collection locations at the Sanxingdui Site.

Soil samples were collected from K3, K4 and K8 in the sacrificial area of Sanxingdui Site. The pit area of K3 was approximately 14 square meters, with a depth of 1.8–2.0 meters. The main filling soil was yellow and brown clay. Unearthed cultural relics included ivory, bronze, jade, pottery, gold, and stone. The soil moisture content of the buried environment was 22.64%, and the pH value was 7.16. The pit area of K4 covered around 8.1 square meters, with a depth of 1.3–1.5 meters. The predominant cultural relics unearthed there were ivory, most of which were found in a burnet state. The nearby fill was stained dark brown by ash. The soil moisture content in this buried environment was 22.08%, and the pH value was 7.18. The pit area of K8 was nearly 20 square meters, with a depth of 1.6–2.0 meters. Among the unearthed cultural relics, bronzes were superimposed under the ivory. The soil moisture content in this buried environment was 20.2%, and the pH value was 7.19 (Figure 1). All three samples were randomly selected from each pit near the ivory site. Three soil samples (K3A, K3B, and K3C) were collected from K3 near the ivory. In K4, three soil samples were selected containing ash (K4A, K4B, and K4C). A control group in an ash-free bottom soil sample (K4D) near the bottom of the buried ivory soil (K4C) was included. Furthermore, three soil samples containing bronze fragments were selected in K8 (K8A, K8B, and K8C) near the ivory. Each sample was sampled three times in parallel, for a total of 30 samples.

To minimize the potential for external contamination, all soil samples were collected using sterile spoons and sealed in pre-sterilized Eppendorf tubes. The collected samples were placed in a liquid nitrogen tank, transported to Sichuan University, stored on dry ice, and sent to Majorbio for subsequent testing.

### 2.2 DNA extraction and MiSeq high-throughput sequencing

Total microbial genomic DNA was extracted from 30 samples using E.Z.N.A.^®^ soil DNA kit according to the manufacturer’s instructions (Omega Bio-tek, Norcross, GA, United States). The quality and concentration of the obtained DNA were determined using 1.0% agarose gel electrophoresis and a NanoDrop2000 spectrophotometer (Thermo Scientific, United States), and stored at −80°C. We used universal primers 338F (5′-ACTCCTACGGG AGGCAGCAG-3′)/806R (5′-GGACTACHVGGGTWTCTAAT-3′) (Liu et al., 2016) and ITS1F (5′-CTTGGTCATTTAGAGGAAGTAA-3′)/ITS2R (5′-GCTGCGTTCTTCATCGATGC-3′) (Gu et al., 2022) to amplify the V3–V4 variable regions of the 16S rRNA gene and the fungal internal transcribed spacer (ITS) regions of the *ITS* gene, respectively. The PCR reaction mixture consisted of 4 μL 5 × Fast Pfu buffer, 2 μL 2.5 mM dNTPs, 0.8 μL (5 μM) each primer, 0.4 μL Fast Pfu polymerase, 10 ng template DNA, and ddH_2_O was added to a volume of 20 μL. The PCR amplification cycle conditions were as follows: initial denaturation at 95°C for 3 min, followed by 35 cycles of denaturation at 95°C for 30 s, annealing at 55°C for 30 s, extension at 72°C for 45 s, and single extension at 72°C for 10 min, and stored at 10°C. The PCR amplification process included a negative control group without template DNA to detect any possible contamination or false positive results. The target band size of the PCR product is correct, the concentration is appropriate, and subsequent experiments can be carried out. Subsequently, the PCR product was extracted from a 2% agarose gel, quantified using a gel extraction kit (AXYGEN, China) according to the manufacturer’s instructions, and purified using a QuantiFluor^™^-ST fluorometer (Promega, United States). The purified amplicons were combined in an equimolar ratio and subjected to paired-end sequencing on an Illumina MiSeq PE300 platform following the standard protocols of Majorbio Bio-Pharm Technology Co., Ltd. (Shanghai, China).

### 2.3 Bioinformatic and statistical analyses

The raw FASTQ files were demultiplexed using an in-house Perl script and subsequently quality-filtered using fastp version 0.19.6 (Chen et al., 2018). Firstly, according to the overlap relationship between PE reads, the paired reads were merged into a sequence. At the same time, the quality of the reads and the effect of the merge were filtered for quality control. According to the barcode and primer sequences at the beginning and end of the sequence, the samples were distinguished to obtain the effective sequence, and the sequence direction was corrected. The merging process was performed using FLASH version 1.2.11 (Magoč and Salzberg, 2011), following the standard criteria. This involved: (i) the reads were truncated at any site receiving an average quality score of <20 over a 10 bp sliding window, and the truncated reads shorter than 50 bp were discarded, reads containing ambiguous characters were also discarded; (ii) only overlapping sequences longer than 10 bp were assembled according to their overlapped sequence. The maximum mismatch ratio of overlap region is 0.2. Reads that could not be assembled were discarded; (iii) Samples were distinguished according to the barcode and primers, and the sequence direction was adjusted, exact barcode matching, 2 nucleotide mismatches in primer matching. Sequences meeting these criteria were aligned with the SILVA database (Quast et al., 2013) and classified into identical operational taxonomic units (OTUs) with a 97% similarity threshold using UPARSE version 11 (Stackebrandt and Goebel, 1994; Edgar, 2013). Chimera sequences were removed in the clustering process and the most abundant sequence for each OTU was selected as a representative sequence. To minimize the effects of sequencing depth on alpha and beta diversity measure, the number of gene sequences from each sample were rarefied. The taxonomies of OTUs were analyzed using Qiime version 1.9.1 and RDP Classifier version 2.13 (Wang et al., 2007), with the SILVA Database 138 for bacteria and UNITE Database 8.0 for fungi, applying a confidence threshold of 70%.

Bioinformatic analysis of the soil microbiota was carried out using the Majorbio Cloud platform.^1^ Rarefaction curves were generated using OTUs with a minimum 97% similarity, and α-diversity indices, including Shannon diversity index, Simpson diversity index, Ace, Chao, and coverage, were computed in MOTHUR v. 1.30.2 (Schloss et al., 2009). The statistical *t*-test was used to detect whether there was a significant difference in the index values between each two groups. Beta diversity analysis was conducted using principal coordinate analysis (PCoA). The UPGMA algorithm was used to construct the tree structure, and the degree of difference in community distribution in different environmental samples was visually presented. Venn diagrams were created in R (v. 3.3.1) and a hierarchical heatmap was generated based on the Bray–Curtis distance using the pheatmap (v. 1.0.8) package for R (v. 3.3.1). Linear discriminant analysis effect size (LEfSe) software was used for LEfSe analysis, with the default filter value of the linear discriminant analysis (LDA) score set to 4.

Raw reads were deposited in the NCBI Sequence Read Archive (SRA) database with accession numbers PRJNA1074310^2^ for bacteria and fungi.

## 3 Results

### 3.1 α-diversity of bacterial and fungal communities

**Figure 2:**
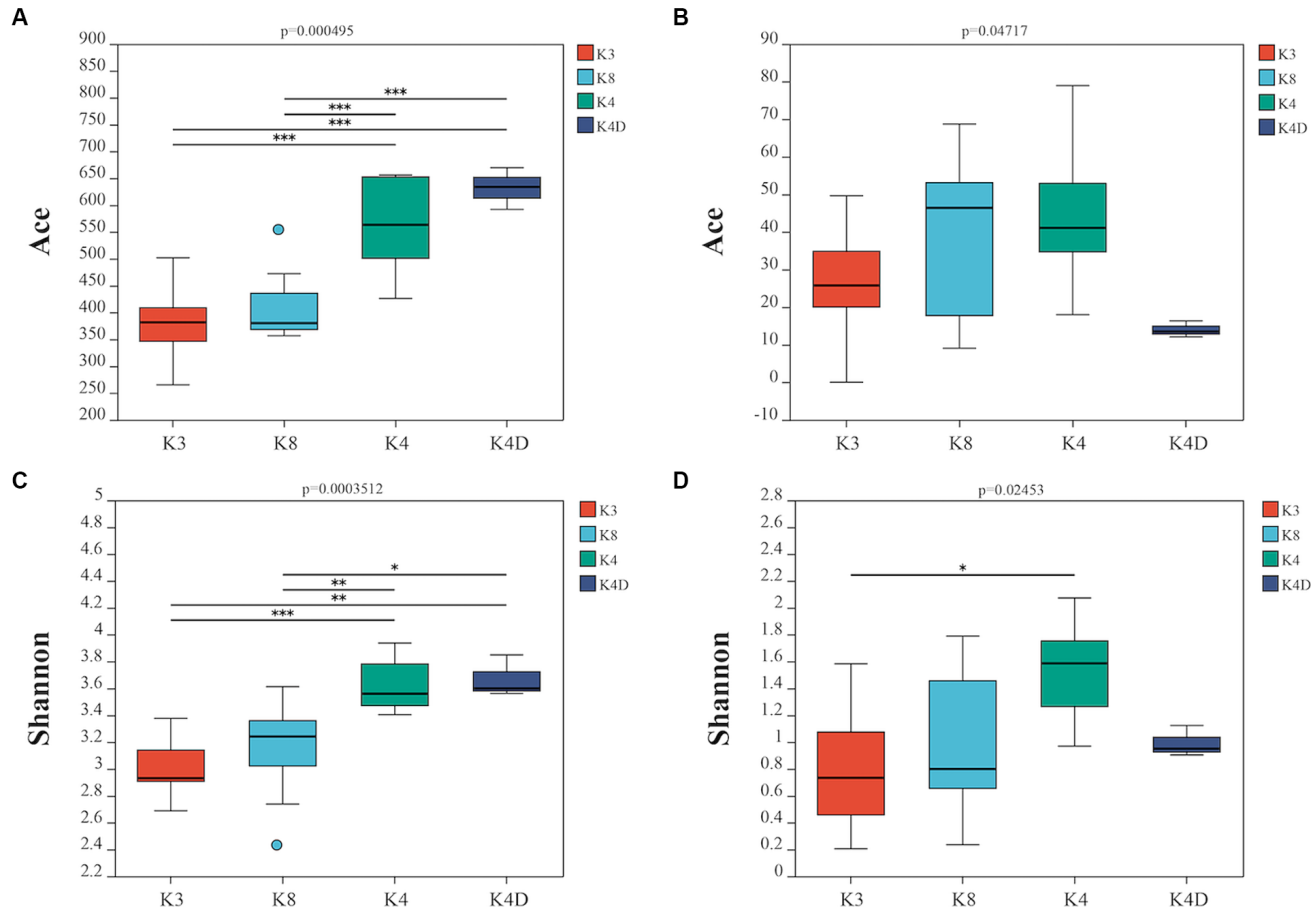
Alpha diversity is represented by the ACE boxplot for bacterial **(A)** and fungi **(B)**, the Shannon boxplot for bacterial **(C)**, and fungi **(D)**.

Using a rarefaction curve is a crucial method to assess data quality since it helps evaluate whether the number and depth of samples can accurately reflect the community structure in the samples (Yan et al., 2021). The rarefaction curves of the high-quality sequencing data for bacteria and fungi were near saturation, indicating that the obtained sequences were sufficient and significant for further analysis (Supplementary Figure S1). For bacteria, there were 2,043,118 valid sequences with an average length of 418 bp, divided into 1,705 OTUs. In the case of fungi, 2,751,884 valid sequences were obtained, with an average length of 243 bp, which were divided into 295 OTUs. Alpha diversity was estimated using the number of OTUs expressed by the Chao 1, ACE, Shannon, and Simpson indices. Additionally, the closer the coverage was to 1, the more accurate the sequencing depth was to the true value. Tables 1, 2 show the results for the bacteria and fungi, respectively. The community species richness (ACE) and diversity (Shannon) of the four groups are shown in Figure 2. For bacterial communities, the highest scores for the ACE and Shannon indices were associated with the K4D group, followed by the K4 and K8 groups, while the lowest scores were observed for the K3 group. This indicated differences in bacterial richness and diversity among the three sacrificial pits. It suggested that the bacterial communities in soil samples from each sacrificial pit exhibited specificity, which might have been related to their geographical locations and soil properties.

**Table 1:** Alpha diversity index of bacteria in soil samples.

Group	Sample	Ace	Chao	Coverage	Shannon	Simpson	Sobs
K3	K3A	430.160	422.007	0.998	3.047	0.103	364
	K3B	394.388	392.884	0.998	3.042	0.097	318
	K3C	302.507	298.712	0.999	2.955	0.108	260
K4	K4A	561.334	549.390	0.998	3.799	0.046	467
	K4B	463.027	449.041	0.998	3.432	0.063	382
	K4C	654.123	635.161	0.997	3.679	0.059	543
K4D	K4D	631.487	619.386	0.997	3.669	0.067	526
K8	K8A	487.154	473.968	0.998	3.433	0.059	407
	K8B	361.125	350.331	0.999	2.842	0.156	324
	K8C	379.818	379.921	0.998	3.141	0.081	324

**Table 2:** Alpha diversity index of fungi in soil samples.

Group	Sample	Ace	Chao	Coverage	Shannon	Simpson	Sobs
K3	K3A	21.710	21.333	0.999	0.750	0.645	21
	K3B	39.257	39.067	0.999	1.275	0.407	39
	K3C	20.169	24.583	0.999	0.424	0.840	24
K4	K4A	49.689	49.278	0.999	1.868	0.233	47
	K4B	53.287	53.033	0.999	1.575	0.276	52
	K4C	25.741	20.833	0.999	1.112	0.402	18
K4D	K4D	13.903	12.833	0.999	0.991	0.431	12
K8	K8A	60.453	59.964	0.999	0.755	0.750	59
	K8B	13.511	12.667	0.999	0.530	0.694	12
	K8C	42.801	43.333	0.999	1.640	0.261	41

For the fungal communities, the order of the ACE index was K4 > K8 > K3 > K4D and the order of the Shannon index was K4 > K4D > K8 > K3. The fungal richness and diversity in Pits 4 and 8 were significantly higher than in Pit 3, possibly due to the environmental characteristics of burned sacrificial pits and those with more bronze artifacts. In such a nutrient-poor environment, fungi may thrive and proliferate better. These findings have certain implications for subsequent ivory conservation efforts.

### 3.2 Bacterial and fungal taxonomic composition

**Figure 3:**
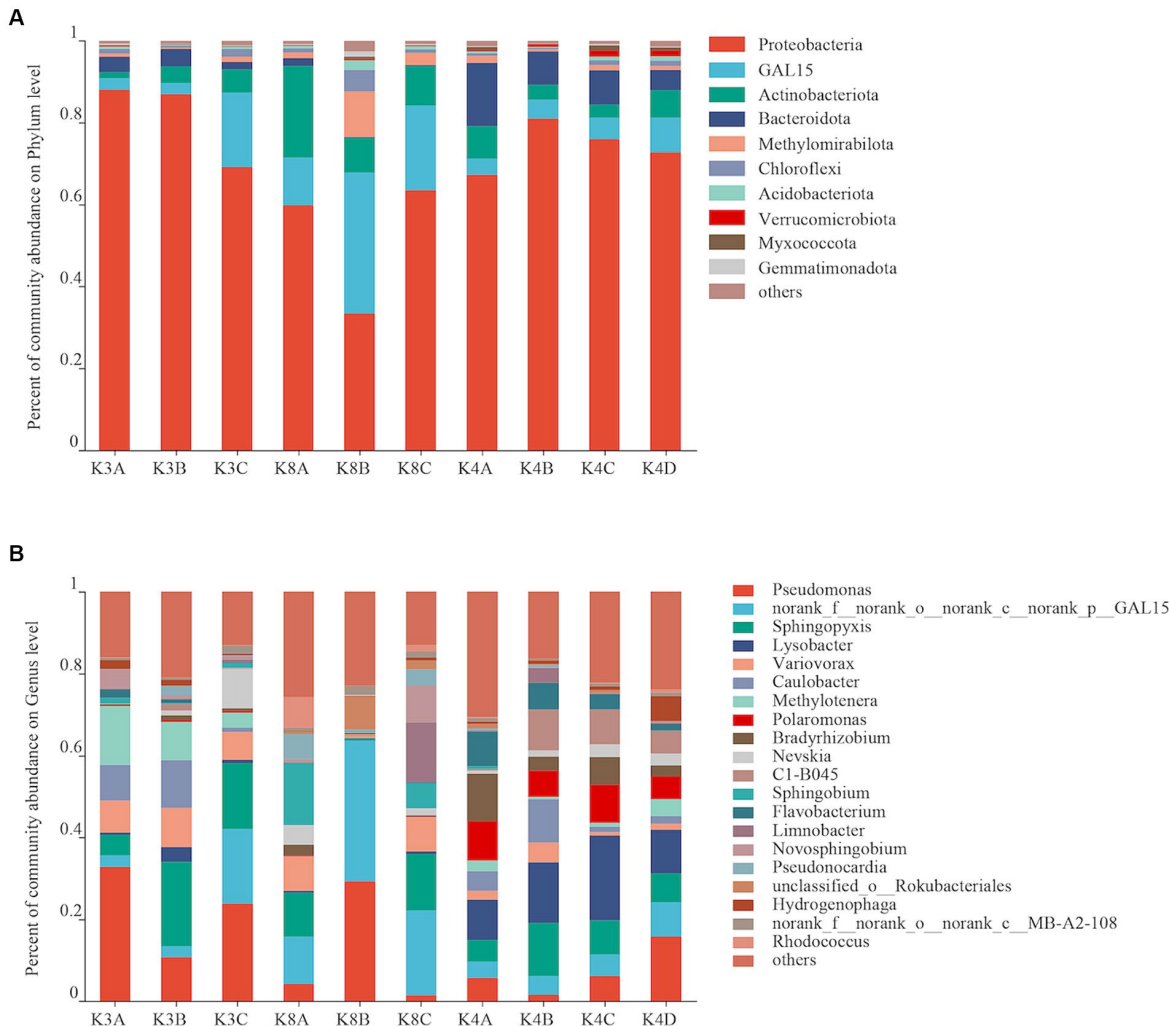
Bacterial community structure at the phylum **(A)** and genus **(B)** levels from all soil samples.

Bacterial OTUs were assigned to 31 phyla, 206 orders, 287 families, 460 genera, and 731 species in the 30 samples. Bacterial communities with an abundance exceeding 2% were categorized as the dominant flora. At the bacterial phylum level, Proteobacteria, GAL15, Actinobacteriota, Bacteroidota and Methylomirabilota emerged as the predominant bacterial phyla, collectively representing 69.88–95.59% of the total sequences (Figure 3A). Proteobacteria was the most dominant phylum, whereas GAL15 and Actinobacteriota were abundant in the K8 group. At the genus level, there were 14 dominant bacterial genera (relative abundance >2%) in soil samples, accounting for 71.65% of all microorganisms (Figure 3B). *Pseudomonas*, *norank_f__norank_o__norank_c__norank_p__GAL15*, and *Sphingopyxis* were the dominant genera in all the groups. The relative abundances of *Lysobacter* and *Polaromonas* were higher in the K4 and K4D groups compared to K3 and K8.

**Figure 4:**
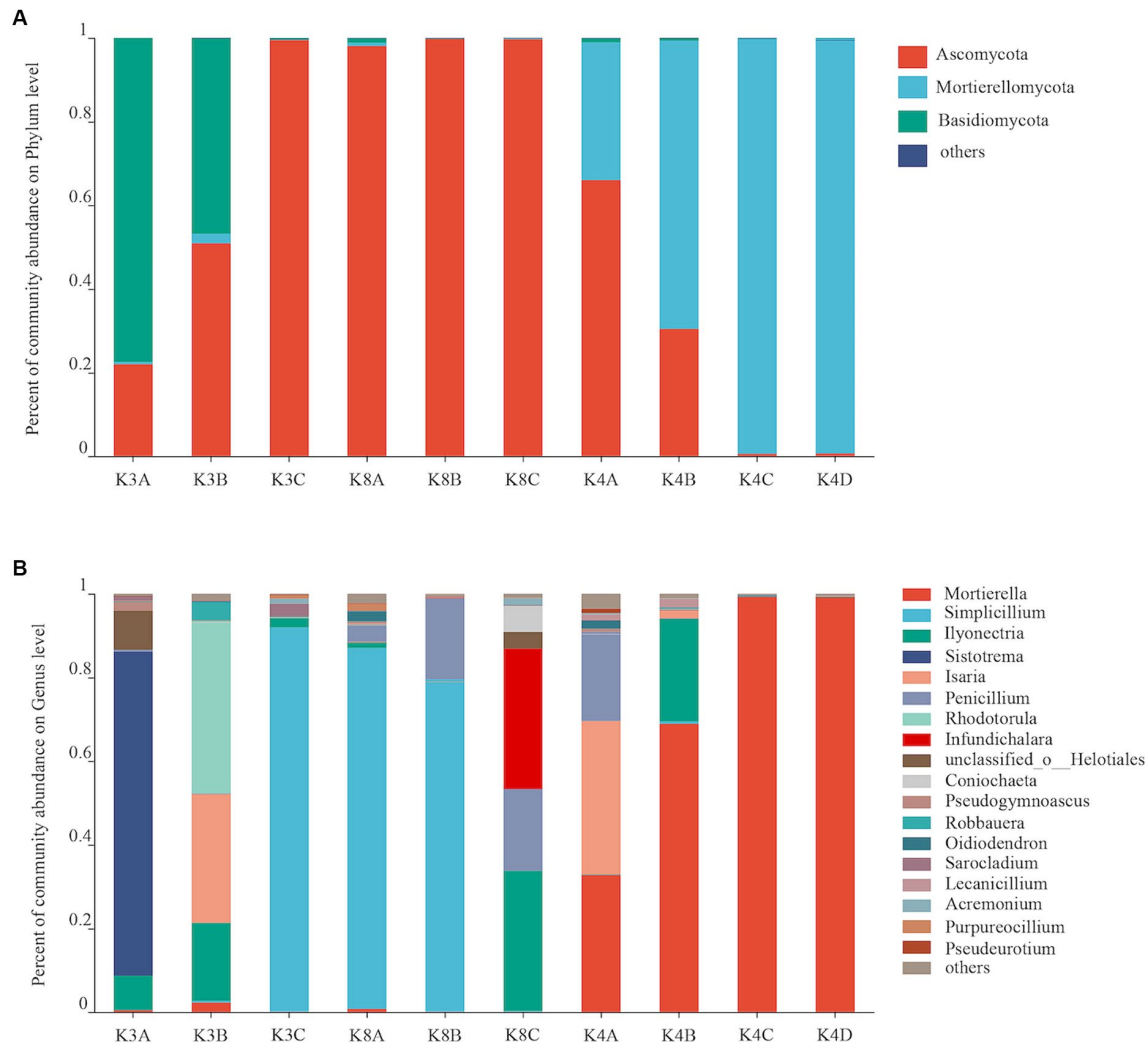
Fungal community structure at the phylum **(A)** and genus **(B)** levels from all soil samples.

The fungal OTUs of all 30 samples were assigned to 5 phyla, 47 orders, 99 families, 151 genera, and 219 species. The fungal communities with an abundance greater than 2% were classified as the dominant flora. There were three dominant fungal phyla (Ascomycota, Mortierellomycota, Basidiomycota), accounting for 56.77–99.96% of all microorganisms (Figure 4A). The relative abundance of Ascomycota was high in most K8 samples, and Mortierellomycota in most K4 samples, respectively. However, Basidiomycota was only predominant in K3. There were eight dominant fungal genera (relative abundance >2%) in soil samples, accounting for 93.96% of all microorganisms (Figure 4B). The most abundant genera were *Sistotrema* in sample K3A; *Simplicillium* in samples K3C, K8A, and K8B; *Isaria* in K4A; *Rhodotorula* in sample K3B; *Infundichalara* in sample K8C; and *Mortierella* in samples K4B, K4C, and K4D.

### 3.3 Bacterial and fungal community structures

**Figure 5:**
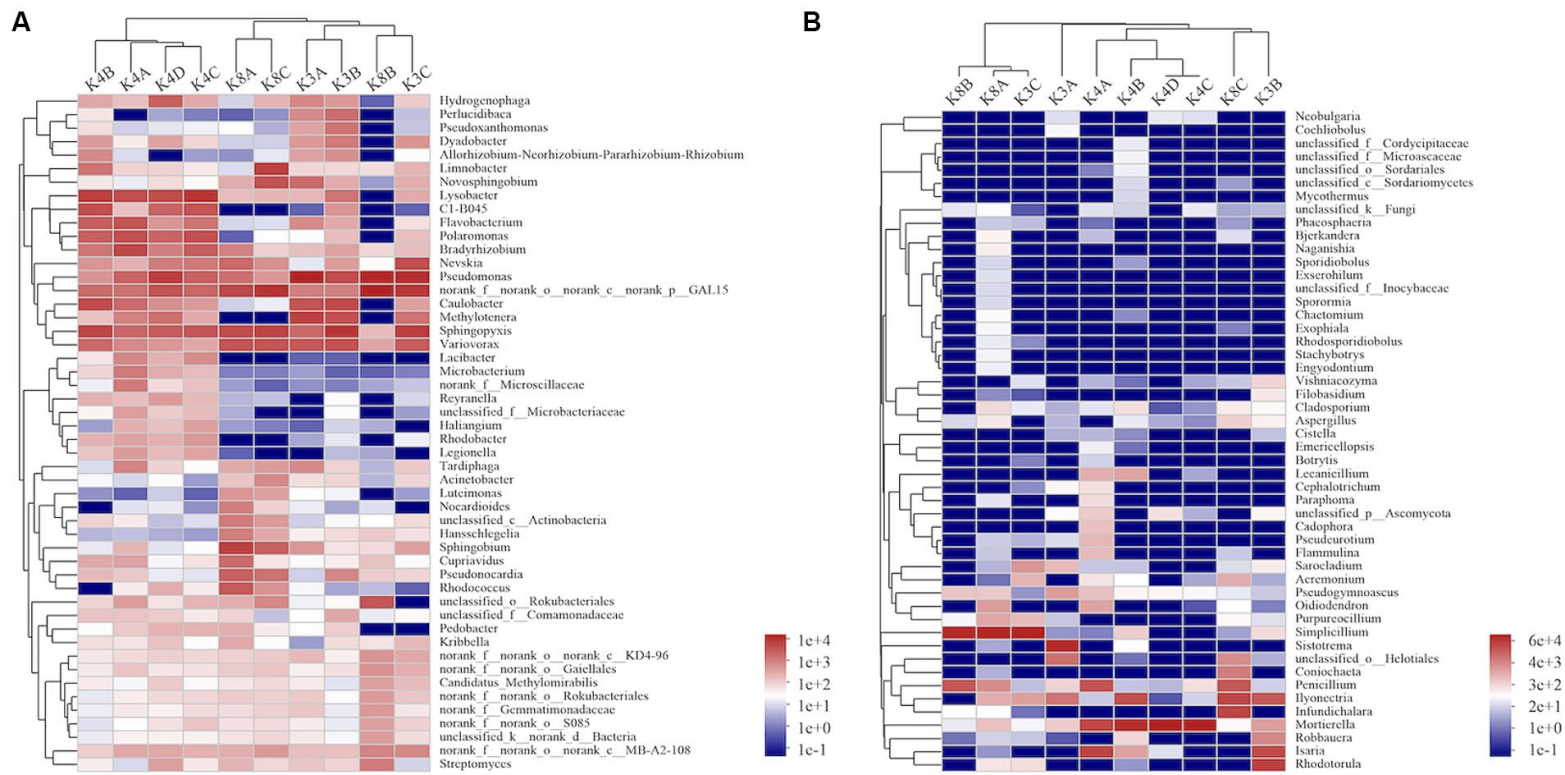
Heatmap hierarchical analysis of the microbial community among the soil samples. **(A)** Bacterial and **(B)** fungal distributions were calculated for the 50 most abundant genera.

**Figure 6:**
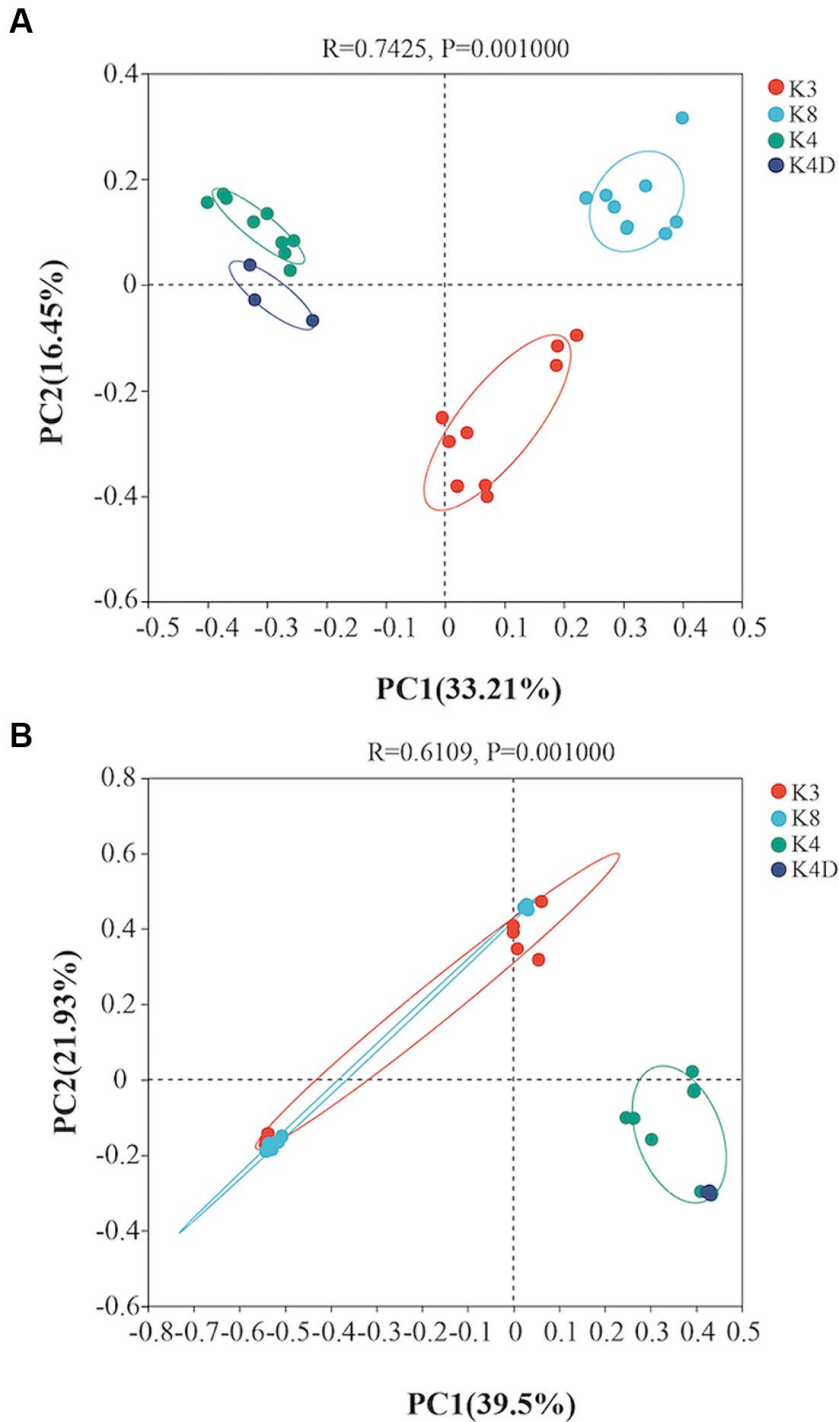
Principal coordinates analysis showing the differences among the **(A)** bacterial and **(B)** fungal communities based on the Bray–Curtis distance.

A heatmap created based on the top 50 bacteria genera clustered into three groups (Figure 5A). The first group consisted of all members of the K4 group, the second group was formed by grouping K8A and K8C, and the third group was primarily composed of K3A and K3B, and grouped with K3C and K8B. There were significant differences in the microbial community structure among soil samples from different sacrificial pits based on the Bray–Curtis distance and PCoA (Figure 6A). Bacterial communities of K4 and K4D were separated from those of K3 and K8 along axis 1.

A heat map based on the 50 most abundant genera of fungi were divided into three groups (Figure 5B). One group comprised of K3C, K8A, and K8B. The second group was composed of all the members of the K4 group, whereas the third group was composed of K3B, K8C, and K3A. The results of PCoA analysis were consistent with the heatmap (Figure 6B), indicating that the fungal communities of samples K4 and K4D were separated from the K3 and K8 groups along PCoA axis 1, and that some fungal communities in the K3 and K8 pits were not clearly distinguished.

### 3.4 Operational taxonomic unit clustering analysis of bacterial and fungal communities

**Figure 7:**
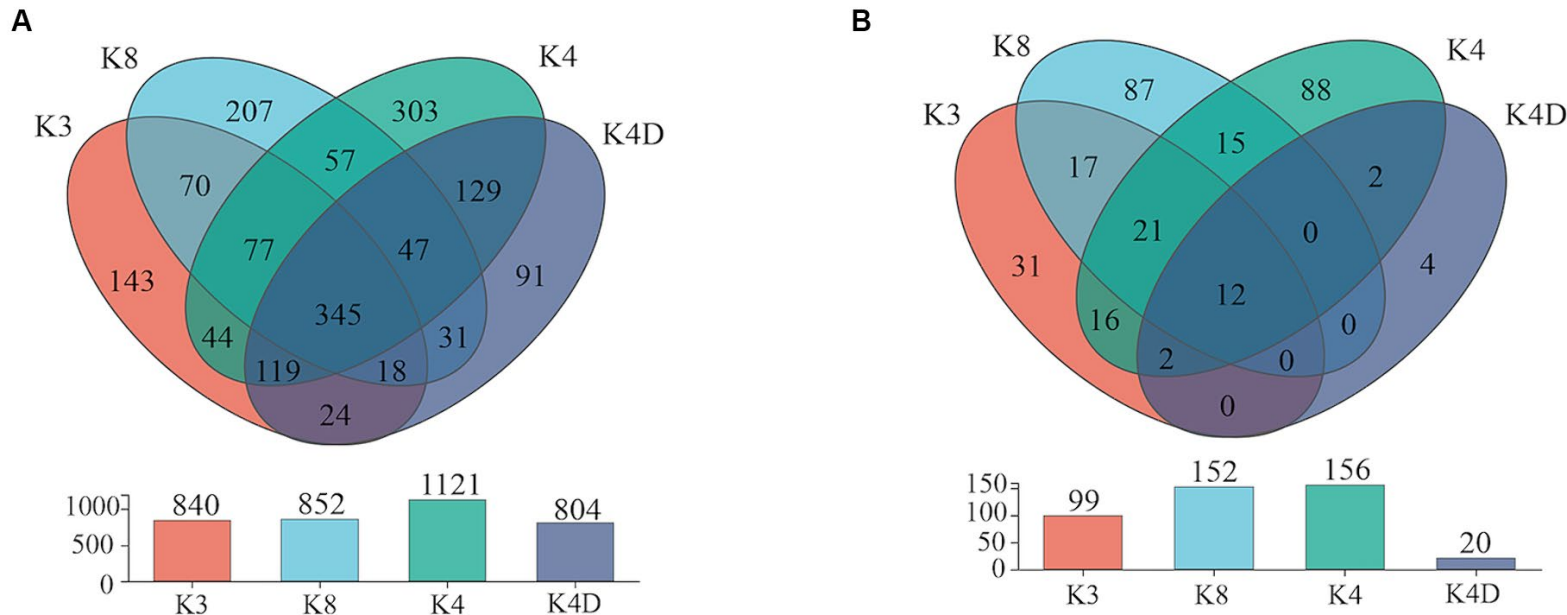
Venn plot of **(A)** bacterial and **(B)** fungal OUT in soil samples.

**Figure 8:**
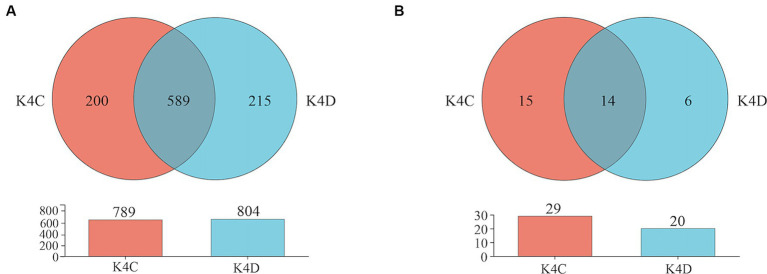
Venn plot of **(A)** bacterial and **(B)** fungal OUT in soil samples from K4.

The 30 bacterial samples were categorized into four groups and ranked as follows: K4 > K8 > K3 > K4D according to the OTU (Figure 7A). Among them, 840, 852, 1,121, and 804 OTUs were obtained in K3, K8, K4, and K4D group, respectively. The number of OTUs shared between the different groups was 345. There were 589 common OTUs between K4C and K4D near the same ivory with or without ash in the soil samples from K4 (Figure 8A). The total number of OTUs in K4D was more than K4C. Proteobacteria dominated the shared OTUs, and the rest of the shared OTUs were affiliated with GAL15, Actinobacteriota, Bacteroidota, Methylomirabilota and Chloroflexi. The unique OUT in K4D was Fusobacteriota (Supplementary Figure S2).

The ranking of the OTU counts for the four groups was K4 > K8 > K3 > K4D for fungi (Figure 7B). The K3, K8, K4, and K4D groups contained 99, 152, 156, and 20 OTUs, respectively. The number of OTUs shared between the different groups was 12. In the soil samples from K4, there were 14 shared OTUs between K4C and K4D, and the total number of OTUs was lower in K4D than that in K4C (Figure 8B). The shared OUTs of the four soil sample groups belonged to Ascomycota, Mortierellomycota, and Basidiomycota, while the unique OUT in K4 was Chytridiomycota (Supplementary Figure S3).

### 3.5 Linear discriminant effect size analysis of bacterial and fungal communities

**Figure 9:**
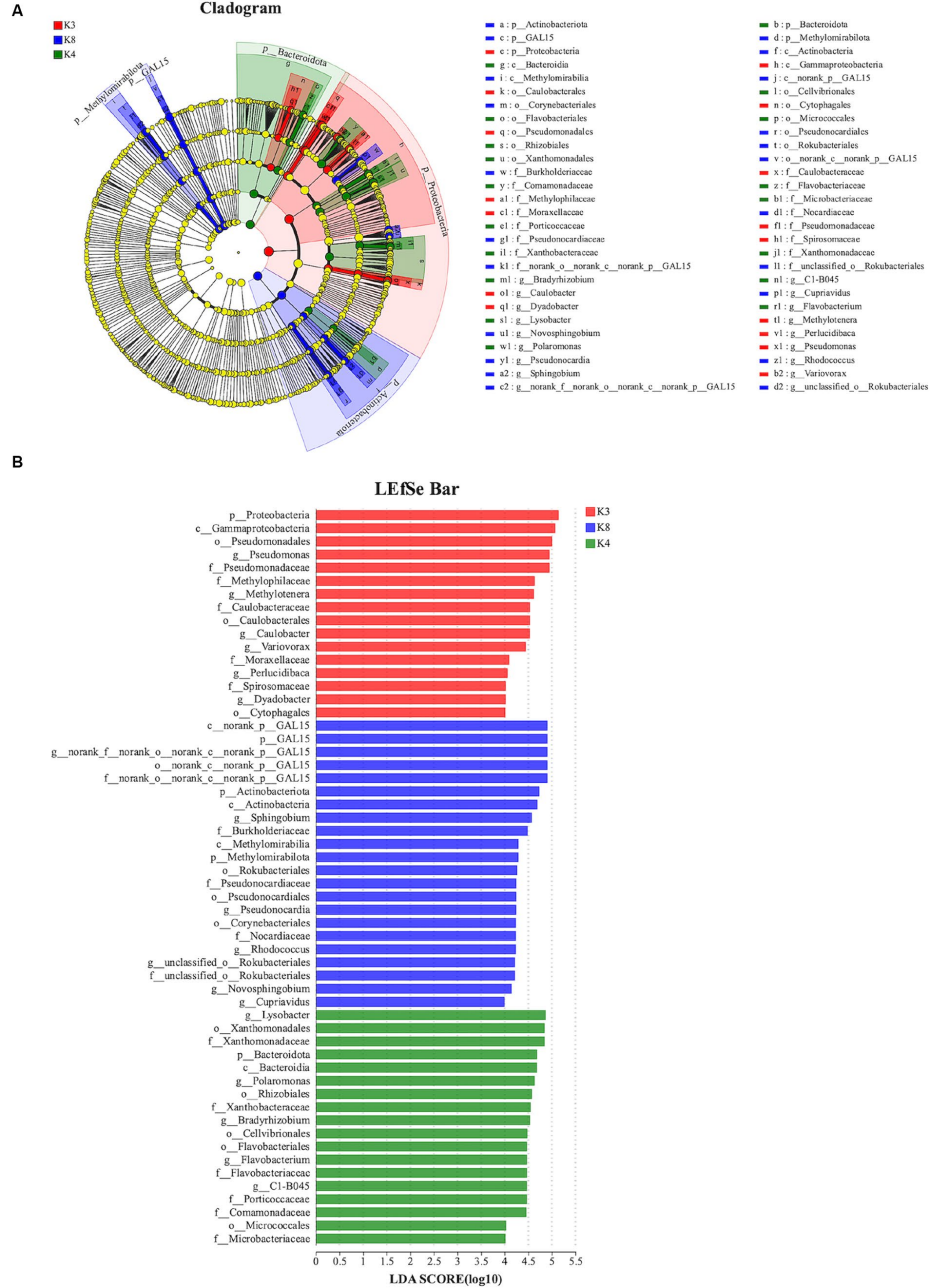
LEfSe analysis of bacterial. **(A)** The development tree diagram reflects the different species at different species levels obtained between different groups. Each node represents a species taxonomy level, and the more abundant the species, the greater the difference. **(B)** Bar chart showing the log LDA score for each species at the taxonomic level. The *X*-axis represents the LDA score (log10), and the *Y*-axis represents the species with significant differences (LDA > 4.0). The larger the LDA score, the greater the effect of species abundance on the difference.

The species showed significant abundance among the three sacrificial pits from the evolutionary branch diagram and bar chart of the LEfSe analysis. For bacteria, 56 bacterial branches exhibited significant differences, with the LDA threshold set to 4.0. The number of biomarkers in the soil of the three sacrificial pits was high, indicating obvious differences among the sacrificial pits. At the phylum level, notable differences were observed in K3 for Proteobacteria; K4 for Bacteroidota; and K8 for GAL15, Actinobacteriota, and Methylomirabilota (Figure 9).

**Figure 10:**
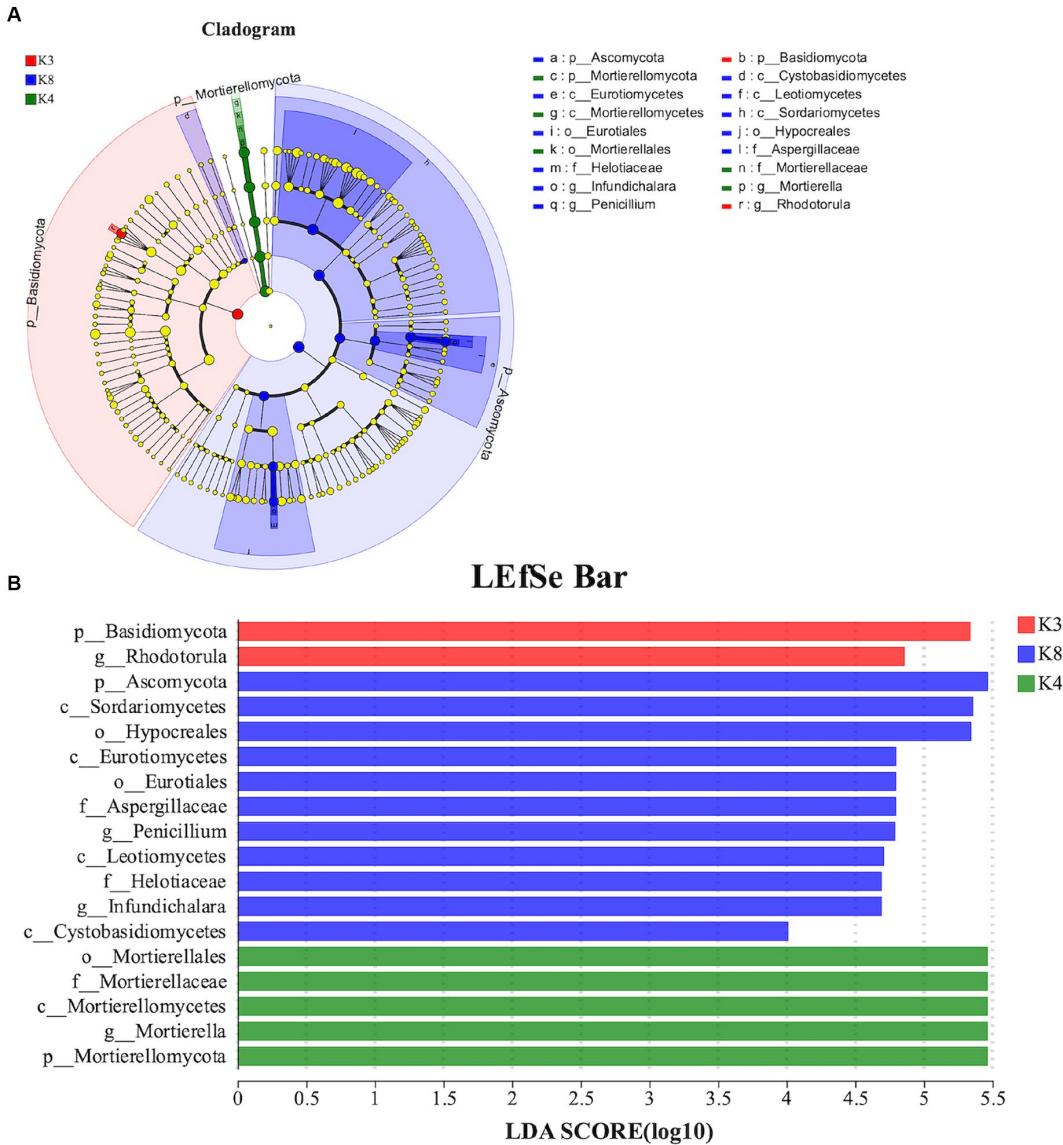
LEfSe analysis of fungal. **(A)** The development tree diagram reflects the different species at different species levels obtained between different groups. Each node represents a species taxonomy level, and the more abundant the species, the greater the difference. **(B)** Bar chart showing the log LDA score for each species at the taxonomic level. The *X*-axis represents the LDA score (log10), and the *Y*-axis represents the species with significant differences (LDA > 4.0). The larger the LDA score, the greater the effect of species abundance on the difference.

For fungi, 18 species exhibited noteworthy differences among the soils of the three sacrificial pits. Specifically, 2 distinct species were identified at K3, 5 at K4, and 11 at K8. There was significant divergence in Basidiomycota in K3, Mortierellomycota in K4, and Ascomycota in K8 at the phylum level (Figure 10). These findings implied that these microbial species could potentially serve as biomarkers of their respective regions, further confirming the differences in soil microbial communities among the sacrificial pits.

## 4 Discussion

### 4.1 Impact of soil microorganisms on buried ivory

Cultural relics often face corrosion and damage caused by microorganisms. Han et al. (2021) analyzed the microbial composition in a desalination buffer and found that the main bacteria and fungi that caused the corrosion of the wooden relics of the Nanhai No.1 shipwreck were *Pseudomonas* and *Cutaneotrichosporon*. Ma et al. (2020) isolated and identified 22 fungal strains from two tombs in the desert of Dunhuang, most of which belonged to the *Penicillium* and *Aspergillus* genera. Chen et al. (2021) studied the microbial diversity in the rocks and soils of the Leshan Giant Buddha and found that *Actinobacteria* and *Proteobacteria* were dominant in the bacterial community, whereas *Ascomycota* and *Basidiomycota* were dominant in the fungal community. The corrosion of different types of cultural relics by microorganisms raises concerns about their preservation of cultural relics and highlights the urgency of protecting precious cultural relics. As a special biological material, ivory has become an important part of art and cultural heritage owing to its hardness, luster, and carving characteristics.

Bacteria are widely recognized as significant contributors to the deterioration of organic cultural relics such as wooden and bone artifacts and inorganic relics such as bronze and iron in burial environments. In the case of ivory relics, bacterial corrosion often occurs through adsorption onto the surface. Metabolic processes produce acidic substances that dissolve the apatite components in the ivory. Additionally, the secretion of various enzymes leads to the degradation of proteins within the ivory, resulting in complete decomposition from the exterior to the interior. The primary phyla of the bacterial community identified in this study were Proteobacteria, GAL15, Actinobacteriota, and Bacteroidota.

Actinobacteriota are widely found in soil, most of which are saprophytic bacteria and have the ability to decompose organic matter. They play an important role in the transformation of organic matter and carbon cycle, and are an important part of humus formation. In addition, they also have efficient secondary metabolic rates, which usually produce and release biochemical substances with specific functions, such as antibiotics, organic acids, polysaccharides, and pigments (Al-shaibani et al., 2021; Duan et al., 2021). These substances may react with the surface of cultural relics, causing corrosion or surface changes, and ultimately causing irreversible damage. Actinobacteriota were identified at various cultural heritage sites, including cave relics, underground tombs, and murals (He et al., 2021; Liu et al., 2022). There is little information on GAL15 bacteria in cultural relics, and further research is needed to elucidate their characteristics. Proteobacteria is a large and diverse bacterial phylum that includes many important soil bacteria. Most Proteobacteria are facultative or obligate anaerobic and heterotrophic or autotrophic organisms. At the same time, they show characteristics of adaptation to a variety of substrates and can survive in a variety of environments, from low acidity to low salt to strongly alkali (Zhou et al., 2020). Bacteroidota are anaerobic bacteria whose anaerobic respiratory metabolites are mainly acetic, isovaleric, and succinic acids (Sun et al., 2022). Ivory artifacts, when exposed to such an environment for an extended period, are susceptible to acid corrosion due to the presence of acidic substances. Another characteristic of the Bacteroidota phylum is its ability to produce spores, which help them survive harsh environments and grow again when environmental conditions are suitable. Its metabolism is vigorous, has a strong degradation effect on complex organic matter and erodes bone relics to a certain extent. Furthermore, the presence of Chloroflexi was also detected in shared OTUs. Chloroflexi play various important ecological roles in natural environments, including organic matter degradation, cycling and transformation, as well as involvement in processes such as soil formation. Since ivory is primarily composed of hydroxyapatite, and some strains within Chloroflexi exhibit phosphatase activity capable of degrading phosphate compounds (Xian et al., 2020), they may participate in the degradation process of ivory. In unique OTUs, Fusobacteriota was found in K4D. Fusobacteriota are typically anaerobes, adapted to low or no oxygen environments (Shanahan et al., 2023), and possess the capability to decompose complex organic matter, thus potentially playing a significant role in the degradation process of ivory.

Among the bacterial genera, the relative abundance of *Pseudomonas* was the highest among all genera, particularly in K3. *Pseudomonas* is a gram-negative non-Bacillus species, and the common representative species are *Pseudomonas aeruginosa*, *Pseudomonas fluorescens*, *Pseudomonas putida*, and so on. Bacteria of this genus also play an important role in the nitrogen cycle and function in nitrogen fixation in the form of NH_3_ (Wu et al., 2024). The fixed NH_3_ is oxidized to nitric acid and nitrous acid under the action of other nitrosation and nitrification microorganisms, which causes acid corrosion of cultural relics. Additionally, research has shown that *Pseudomonas aeruginosa* has the ability to dissolve both inorganic and organic phosphate (Miller et al., 2010). Therefore, *Pseudomonas* may play a key role in ivory corrosion. The distribution of *Sphingopyxis* in each sample is relatively uniform. *Sphingopyxis* is an important biodegradable bacterium with strong degradation ability, metabolic diversity, and adaptability to the environment. These capabilities may have caused the organic matter in cultural relics to break down. The genus *Lysobacter* exhibited a relatively high abundance in both K4 and K4D. As saprophytic microorganisms, they are widely distributed in soil and rely on the degradation of organic matter and interactions with other microorganisms for survival. These strains not only effectively decompose organic matter but also have the ability to produce brown water-soluble pigments (Guanghai, 2011). It is worth noting that as the strains age, the secretion of pigment increases, indicating their potential role in the corrosion of ivory.

In addition to bacteria, fungi commonly cause the degradation of cultural relics. The most significant feature of fungi is their developed mycelia, which can damage cultural relics through puncture of the mycelia at an opportune place (Björdal, 2012). At the same time, fungi can also secrete acidic substances, enzymes, and colored substances. These metabolites can corrode the surfaces of cultural relics, resulting in irreversible damage to their value. When the enzyme solution released by fungi adheres to the surface of ivory, it leads to a difference in oxygen concentration around the ivory. This difference accelerates the occurrence of electrochemical reactions, thereby speeding up the corrosion process of ivory. The distribution of fungi in the three pit soils was random. The dominant fungal phyla can all produce spores, leading to contamination on the surface of ivory. The Ascomycota phylum forms ascus and ascospores through sexual reproduction, thereby causing plant decay and a series of diseases. Meanwhile, the Basidiomycota phylum, composed of multicellular organisms with septate hyphae, produces basidia as spore-bearing structures and can also generate external sexual spores called basidiospores. As one of the highest phyla in the fungal kingdom, Basidiomycota is widely distributed with numerous species, capable of causing diseases in forests and plants, resulting in wood decay, and even promoting organic matter decomposition (Ludley and Robinson, 2008). In the OTU clustering analysis, we identified Chytridiomycota as a unique OTU in K4. Chytridiomycota are typically saprophytic, releasing enzymes to decompose decaying organic matter in the environment and then obtaining nutrients through osmotic absorption (Midgley et al., 2006). In the process of ivory degradation, Chytridiomycota may intervene and participate in breaking down ivory tissue into simpler compounds, thereby promoting the cycling and decomposition of organic matter. Among all the fungal genera, *Mortierella* had the highest relative abundance. It usually exists in the form of saprophytic bacteria in soil, animal bones, or other organic matter with strong degradation ability (Li et al., 2023a). Therefore, this fungal strain may damage ivory composition and can be considered a potential corrosion threat. Therefore, although the composition of various microorganisms in the soil may vary slightly, dominant microorganisms may affect the strength and coloration of ivory to varying degrees, necessitating timely measures for disease prevention and control.

In general, the microbial communities in soil synergistically facilitated the decay and damage of ivory through various means. Bacteria and fungi played crucial roles in this process. They secreted various enzymes such as cellulases, amylases, and proteases, breaking down the complex organic compounds in ivory into smaller molecules, thus providing microbial communities with more readily utilizable energy sources. At the same time, they could also produce organic acids or inorganic acids such as acetic acid, citric acid, and sulfuric acid, which could lower the pH of the ivory surface, leading to oxidation and corrosion. These intricate enzymatic mechanisms and metabolic pathways collaborated to accelerate the decay and damage of ivory in the burial environment. Therefore, in the conservation strategy for ivory, potential risks can be identified early by regularly monitoring the microbial communities on the surface of artifacts and in the surrounding environment. For instance, genetic sequencing techniques can be employed to identify microbes and assess their potential harm to ivory, thus effectively safeguarding precious artifacts such as ivory from microbial damage.

### 4.2 Impact of ash on the diversity of soil microbial communities in K4 soil

For bacteria, the sample with ash had fewer OTUs than the sample without ash (K4C < K4D) according to operational taxonomic unit (OTU) clustering analysis. In the case of fungi, K4C and K4D shared 15 OTUs. Notably, the unique OTU count in K4C was more than twice that in K4D, indicating a higher richness of fungal communities in the ash-containing soil samples resulting from combustion. After high-temperature treatment, the pH, organic matter content, nutrient composition, structure, and redox state of the soil changed. Furthermore, the inherent characteristics of the microorganisms contribute to noticeable differences in their distribution within the soil (Panico et al., 2020). High-temperature incineration can result in the inactivation or death of certain bacteria, particularly those that are sensitive to temperature fluctuations. This process also results in ash production, thereby increasing the soil pH, creating an alkaline environment, and lacking adaptive nutrients, all of which inhibit bacterial growth and reproduction. Compared to bacteria, fungi may exhibit greater adaptability to conditions arising from high-temperature incineration. The spores or fruiting bodies of certain fungi are more resilient in post-incineration environments, which facilitates rapid reproduction. They demonstrate a superior ability to adapt to diverse environments compared to bacteria owing to the more complex cell walls of fungi. In environments with high alkaline soil and the release of toxic substances, fungi may adapt by regulating their own physiological activities, such as producing specific enzymes to degrade toxic substances (Kües, 2015). Furthermore, some fungi possess saprophytic capabilities that allow them to decompose organic matter into energy. This enables them to locate and utilize organic matter residues in ash-containing soils. They may also decompose organic components in ivory, leading to its decay and degradation. Therefore, measures need to be taken to control and prevent fungal growth and activity when protecting ivory artifacts, such as adjusting environmental conditions, using protective coatings, and regular cleaning, to reduce fungal damage to artifacts.

PCoA analysis also revealed that the bacterial and fungal community structures in Pit 4 were significantly different from the other two pits. As discussed, burning could potentially alter the physical, chemical, and biological properties of the soil, consequently impacting the composition and structure of microbial communities. Additionally, burning might also modify the burial conditions of the ivory, such as temperature, humidity, and aeration, thereby affecting the distribution and activity of microorganisms in the soil. Furthermore, human activities such as agricultural practices and industrial pollution could also influence the soil environment and microbial community structure. Due to the close correlation between the preservation status of ivory and changes in soil environment as well as microbial community structure, it is necessary to comprehensively consider multiple aspects when designing effective ivory conservation measures.

The ash layer adsorbs a large amount of water, so that the soil moisture content reaches 60%, which is 40% higher than the normal soil moisture content. It can effectively maintain soil moisture to a certain extent owing to the strong water retention properties of ash. Ash that is mixed or buried in soil can improve soil water retention and prevent rapid water loss. Consequently, the soil in K4C had a higher water content than that in K4D. Moisture directly affects the growth and metabolic activities of microorganisms. Diversity analysis showed that there was no significant difference in bacterial community diversity between K4C and K4D, indicating that water had a relatively small effect on bacterial community diversity. In contrast, the fungal diversity in K4C was higher than that in K4D. This suggests that the presence of ash increased the diversity and complexity of the fungal community and indicates that the microbial species near the ivory were more abundant, highlighting the significant impact of microorganisms on the ivory.

### 4.3 Impact of bronze relics on microbial communities in the K8 environment

After a long burial period, many bronze pieces in K8 gradually corroded, produced rust, and dissolved in the soil. The rust infiltrated the ivory through the soil, resulting in the unearthed ivory at K8 to appear green. The copper content in the K8 soil sample was 4.2 times higher compared to K3 (Deng et al., 2023), indicating a more severe impact of bronze artifacts on K8 soil. This obvious difference in Cu content may lead to the destruction of the soil particle structure, which in turn affects soil permeability and water retention. As discussed, the ivory unearthed from the Sanxingdui site is mainly composed of hydroxyapatite. However, its crystal structure contains certain defects, which may cause ivory to be easily adsorbed by heavy metal ions in the environment, thereby causing structural damage and dissolution. Simultaneously, elevated concentrations of rust may have inhibited some enzyme activities in the soil, affecting the degradation of organic matter and the release of nutrients in the soil, resulting in changes in the microbial community structure.

Compared with other sacrificial pits, the GAL15 content in Pit 8 was the highest at the phylum and genus levels, indicating that GAL15 adapted to grow in a copper-infected oligotrophic soil environment, which is consistent with previous soil studies (Frey et al., 2021; Yin et al., 2023). Linear discriminant analysis effect size analysis of high-throughput sequencing data revealed significant variations in the dominant flora among the different sacrificial pits, with K8 exhibiting significantly greater differences in species compared with the other pits. Additionally, K8 soil was enriched in fungal phyla, of which Ascomycota was the main fungal group. Certain fungi within Ascomycota are acid-producers, particularly molds and *Aspergillus* fungi, which can generate organic acids, such as citric acid and oxalic acid through metabolic processes (Li et al., 2024). Ascomycota can also degrade polysaccharides and proteins via enzyme secretion. This phenomenon is likely to have a lasting impact on ivory.

### 4.4 Strategies for the prevention and control of harmful microorganisms

As an effective biological control method, antimicrobial agents have the potential to prevent and control microbial diseases, especially for microorganisms that cause quality damage to ancient ivory. However, the feasibility of antimicrobial therapy in cultural heritage preservation hinges on the materials and extent of damage to the artifacts. Some artifacts may be resistant to antimicrobial agents and require specific treatment regimens to avoid additional damage. Additionally, the effectiveness of the treatment depends on the effect of the selected drugs on the target microorganisms, and microorganisms on ancient cultural relics may have developed resistance to traditional antibiotics. Ethical considerations, including factors that may impact the originality of artifacts and their environmental surroundings, must also be considered when employing antimicrobial therapy. Therefore, while antimicrobial therapy holds promise in cultural heritage preservation, it requires careful consideration to ensure artifacts and their environments remain unharmed, while respecting the value and authenticity of historical cultural heritage.

In the subsequent studies, the potential disease microorganisms can be cultured and isolated, and then the screening and effect evaluation of bacteriostatic agents can be carried out. These data will provide a scientific foundation to prevent and control microbial diseases affecting ancient ivory unearthed in Sanxingdui, so as to further promote the protection and preservation of ancient ivory cultural heritage. In order to cultivate and isolate potential pathogenic microorganisms, it is necessary to use advanced culture techniques and separation methods. Screening of antimicrobial agents requires high-throughput screening, molecular docking, and other technologies. The optimization of methods in the practice of cultural relics protection is a continuous process, which requires continuous practice and summary of experience, and interdisciplinary cooperation to solve practical problems. To advance this goal, specific conservation initiatives need to be developed, including scientific research and conservation plans, cultural heritage conservation policies and regulations, education and public awareness initiatives, as well as technological innovation and application. Additionally, establishing broad partnerships is crucial, including international organizations and institutions, academia and professional bodies, as well as the general public and non-governmental organizations, to collectively promote the protection and inheritance of ancient ivory cultural heritage. In general, these research directions require long-term investment and interdisciplinary cooperation to solve microbial-related problems in the protection of cultural relics, and continuously optimize and improve the methods and technologies for the protection of cultural relics.

Consequently, studying the buried ivory soil microorganisms at the Sanxingdui site is just one aspect of cultural heritage preservation. However, it also presents higher demands for the scientific and meticulous excavation of archaeological sites. Simultaneously, the scientific and meticulous excavation of archaeology provides a substantial foundation for on-site conservation and technological analysis, collectively driving the thriving development of cultural heritage preservation.

## 5 Conclusion

High-throughput sequencing identified the structure and diversity of bacterial and fungal communities in buried ivory soil samples collected from the Sanxingdui Site. Dominant bacterial phyla included Proteobacteria, GAL15, Actinobacteriota, Bacteroidota, and Methylomirabilota. Dominant fungal phyla were represented by Ascomycota, Penicillium, and Basidiomycota. The presence of these microbial taxa may have implications for ivory preservation, including accelerating organic matter decomposition, inducing surface corrosion, and affecting microbial ecological balance. Significant differences in microbial presence were observed among different sacrificial pits. The divergence in microorganism composition is closely linked to the distinct burial environments resulting from the unique filling methods and cultural relic accumulation practices in each sacrificial pit. Therefore, studying microbial communities in ivory burial environments is crucial in archaeology and cultural heritage preservation for developing appropriate conservation measures and management strategies.

## Data availability statement

The datasets presented in this study can be found in online repositories. The names of the repository/repositories and accession number(s) can be found at: https://www.ncbi.nlm.nih.gov/sra, PRJNA1074310.

## Author contributions

SS: Formal analysis, Methodology, Writing – original draft, Writing – review & editing. ZXu: Formal analysis, Writing – original draft. MR: Data curation, Writing – review & editing. SL: Resources, Writing – review & editing. ZXi: Writing – review & editing. YL: Methodology, Writing – review & editing. YT: Data curation, Formal analysis, Methodology, Writing – original draft, Writing – review & editing.

## Footnotes

^1^https://cloud.majorbio.com

^2^https://www.ncbi.nlm.nih.gov/sra
