# Supplementary figures and images for: Identification of microbial diversity in buried ivory soil at the Sanxingdui site in Guanghan City, China, using high-throughput sequencing
Source: Front Microbiol. 2024 May 30;15:1384650. doi: 10.3389/fmicb.2024.1384650 (PMC11169624; doi:10.3389/fmicb.2024.1384650)

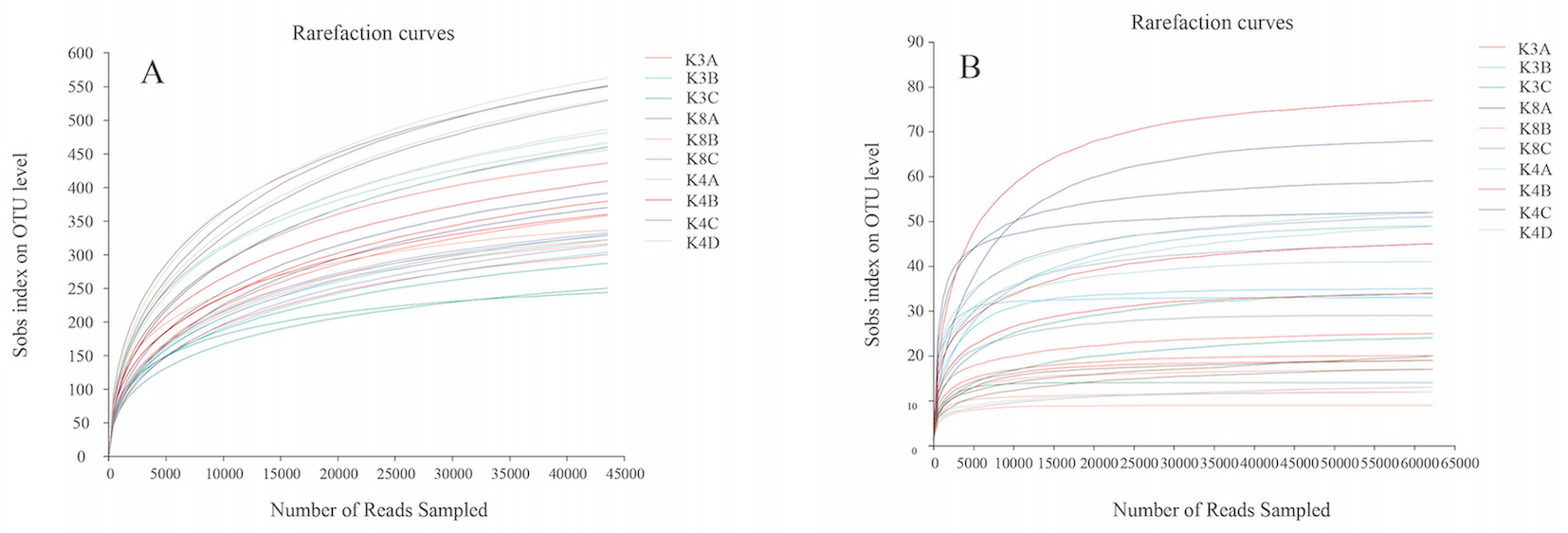

Supplement: Supplementary file 1 [file Image_1.TIF]

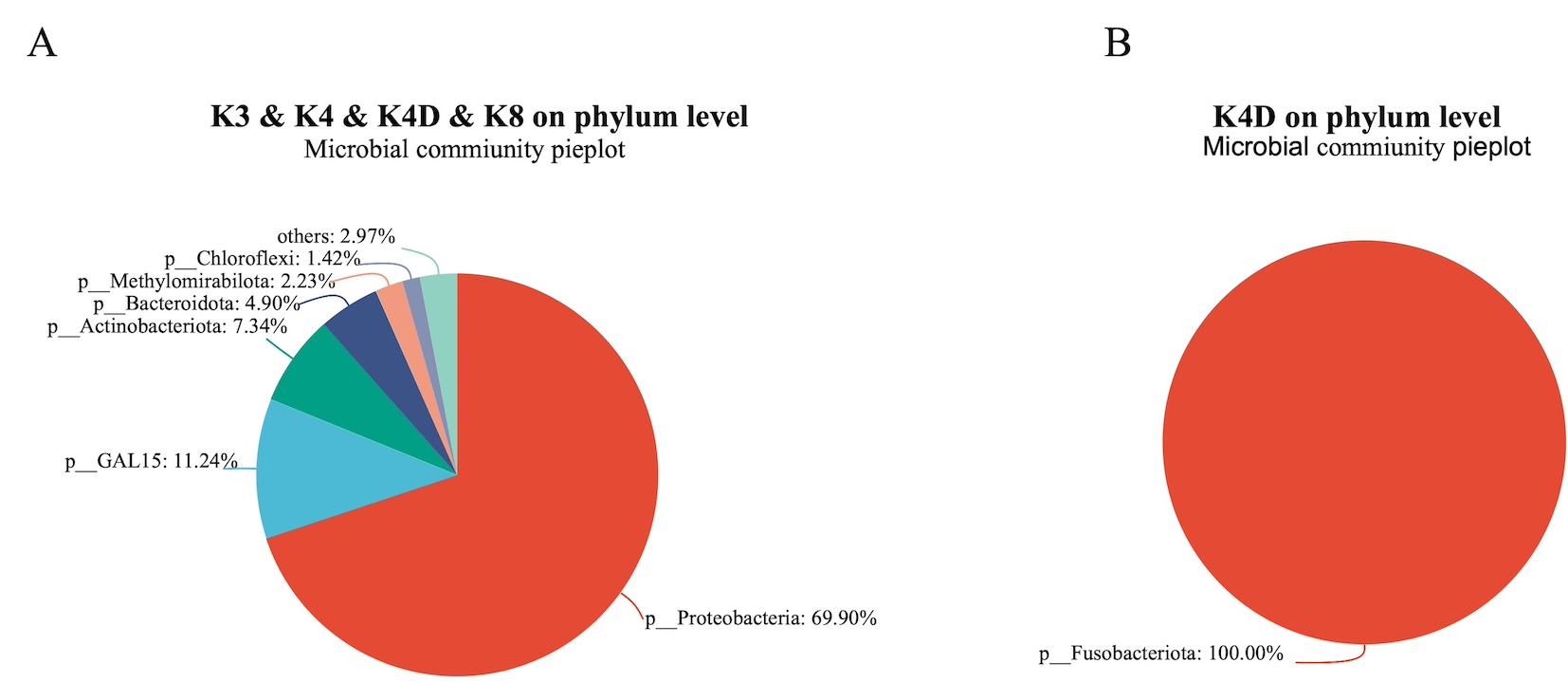

Supplement: Supplementary file 2 [file Image_2.JPEG]

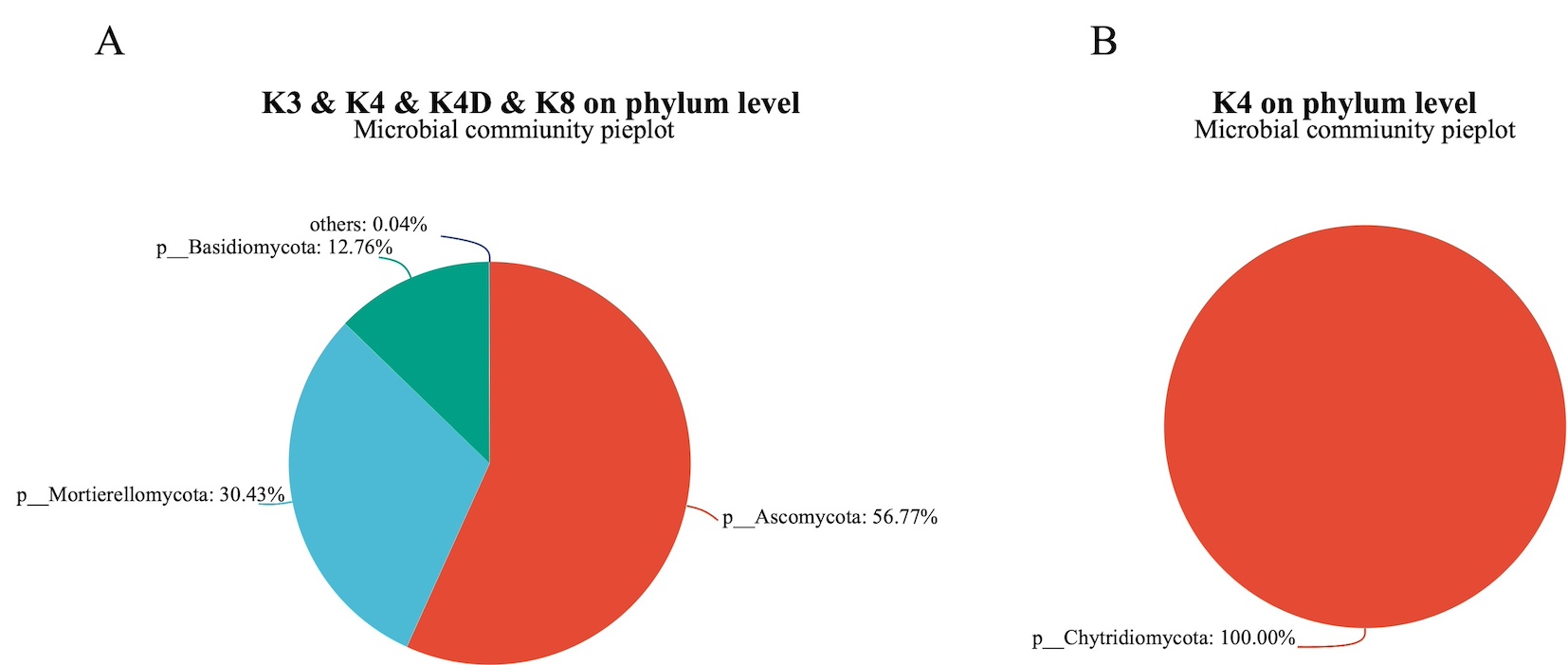

Supplement: Supplementary file 3 [file Image_3.JPEG]
